# Reliability of a smartphone goniometer app compared with traditional goniometer for measuring passive motion at the first metatarsophalangeal joint

**DOI:** 10.1186/1757-1146-8-S2-P12

**Published:** 2015-09-22

**Authors:** Georgie Hales, Richard Keating, Nicola Bear, Katie Warren, Simon Otter

**Affiliations:** 1 University of Brighton, School of Health Sciences, 49 Darley Road, Eastbourne, BN20 7UR, UK

## Background

Adequate sagittal plane motion of the first metatarsalphalangeal joint (1^st^ MTPJ) is important during normal gait and goniometric measurement is commonly used as a diagnostic and outcome assessment tool for 1^st^ MTPJ pathology. We aimed to determine the intra and inter-rater reliability of a universal plastic goniometer and a smartphone application when measuring dorsiflexion at the 1^st^ MTPJ.

## Method

A double-blind repeated measures design was used (three raters, one observer) to measure 1^st^ MTPJ dorsiflexion in both feet of 32 healthy volunteers (64 data sets) using a pre-defined measurement protocol. Reliability of the smartphone app (Dr. Goniometer v1.8 installed on an iphone 4s) and a standard universal goniometer was assessed using Interclass correlation coefficients (ICCs) with 95% confidence intervals.

## Results

Both instruments demonstrated excellent intra-rater reliability (ICC> 0.75), with moderate to good inter-rater reliability:

Universal Goniometer

Inter-rater reliability ICC 0.693 (95% CI 0.580 – 0.788)

Smartphone app

Inter-rater reliability ICC 0.708 (95% CI 0.597 - 0.799)

## Conclusions

Moderate to high intra and inter-rater reliability of passive 1^st^ MTPJ motion can be achieved with traditional and smartphone-based goniometric measurement. Smartphone applications may provide a slightly higher reliability, but devices should not be used inter-changeably as significant variations in measurement between devices may occur.

